# Characteristics and health care costs in patients with a diagnostic imaging for low back pain in Switzerland

**DOI:** 10.1007/s10198-021-01397-8

**Published:** 2021-10-30

**Authors:** Stefania Di Gangi, Christophe Bagnoud, Giuseppe Pichierri, Thomas Rosemann, Andreas Plate

**Affiliations:** 1 Institute of Primary Care, University and University Hospital Zürich, Pestalozzistrasse 24, 8091 Zürich, Switzerland; 2 Groupe Mutuel, Martigny, Switzerland

**Keywords:** Low back pain, Characteristics, Diagnostic imaging, Economic burden, Health care costs, I13

## Abstract

Low back pain (LBP) is one of the most common musculoskeletal disorders worldwide and a frequent cause for health care utilization with a high economic burden. A large proportion of diagnostic imaging in patients with LBP is inappropriate and can cause more harm than good, which in turn can lead to higher health care costs. The aim of this study was to determine characteristics and health care costs for patients with a diagnostic imaging for LBP in Switzerland. Groupe Mutuel, one of the biggest health care insurance companies in Switzerland and covering approximately 12% of the population, provided data for this analysis. Patients were identified by diagnostic imaging for the lumbar spine in 2016 or 2017. The study period was 2015–2019, that is one year before and two years after the year of imaging. Regression analysis models were used to identify patient variables associated with higher health care costs. A total of 75,296 patients (57% female, mean age: 54.5 years) were included into the study. Magnetic resonance imaging was the most commonly used diagnostic method (44.3%). Patients generated annual mean health care costs of 518,488,470 CHF (466,639,621 Euro) in the whole observation period; 640 million CHF (576 million Euro) in the index year. Overall, costs for LBP patients were 72% higher compared with the costs of no LBP patients. Our findings confirm the economic burden of LBP and highlight the importance of ongoing efforts to improve prevention, diagnostics and patient care in patients with LBP.

**Supplementary Information:**

The online version contains supplementary material available at 10.1007/s10198-021-01397-8.

## Introduction

Acute non-specific low back pain (LBP) is one of the most common musculoskeletal disorders worldwide [1–3]. With a lifetime prevalence of up to 80% [4, 5], LBP entails a high social burden. Despite the individual burden of affected patients, health care costs of LBP are of particular interest and various studies using quite different methodological approaches report estimates of costs of patients with LBP [6]. Due to the different health care systems and cultural factors, health care costs may differ greatly between countries [3]. Switzerland is one of the countries with the highest health care spending in the world [7] and a study published in 2011 estimated the total yearly costs of LBP in Switzerland to 6.6 Billion Euro, which corresponds to 2.3% of the national gross domestic product (GDP) [8].

Radiological imaging is an important cost driver in the context of LBP [9]. Even if not always necessary in many acute LBP cases, radiological imaging is a common part of the patient workup [10]. The images themselves cause health care costs and incidental findings, requiring further investigations, generate further health care costs. This is one reason, why various guidelines promoting good clinical practice are available [10] and the recommendation of avoiding unnecessary radiological imaging is one of the most prominent messages of both international and national choosing wisely campaigns [11, 12]. In addition, a range of further comorbidities like depression, anxiety or sleep disorders has shown to be associated with LBP [13], thus contributing to increased health care costs in this population.

This combination of high LBP-associated costs and concomitant high prevalence, illustrates that monitoring health care costs, and not only LBP specific costs, and identifying patients with a risk of increasing costs is of utmost importance. Analyses that specifically report the total health care costs of patients with diagnostic imaging for LBP, and not only the specific LBP-associated costs, are lacking. The fact that a large proportion of diagnostic imaging is inappropriate also highlights the importance of such analyses. With this knowledge, health care providers, insurance companies and official health authorities can improve strategies and interventions fostering prudent patient care, which in turn can contribute to limit rising health care costs.

The objective of the present study was to determine patient characteristics and actual data on the health care costs for patients with a diagnostic imaging for LBP in Switzerland using health claims data provided by a national health insurance company. In addition, we aim to determine the incremental health care costs due to LBP.

## Material and methods

### Study design and database

In this retrospective observational study, we analysed health care data provided by Groupe Mutuel (GM). GM is one of the largest health insurance company in Switzerland, covering approximately 12% of the total Swiss population in 2016 and 2017. GM insures patients in all parts of Switzerland, although a higher proportion of the population is insured in the French-speaking part of Switzerland (up to one-third of the population). Due to applicable regulations provided by GM, we analysed a random selected subset of around 92% of all identified patients.

### Inclusion criteria and definitions

We identified patient costs of LBP using tarif medical (TARMED) codes coding imaging methods of the spine as a surrogate for LBP. TARMED codes were used to code all medical procedures for reimbursement in the Swiss outpatient setting. We included patients by (i) specific TARMED codes for the lumbar spine (39.0150 and 39.0155 [x-ray of the spine, first image, and further images]), and (ii) unspecific TARMED codes for the spine (39.4100 [computed tomography (CT) of the spine]), and 39.5060 [magnetic resonance imaging (MRI) of the spine]). We included all patients ≥ 18 years of age.

To prevent incorrect inclusion of patients based on non-specific codes, we excluded patients from the analysis if they had an unspecific imaging code (CT or MRI) in combination with a specific TARMED code for an adjacent anatomic region (for example combined TARMED codes for CT of the spine and CT of the skull). These combinations, in fact, could indicate that the pathology was not exclusively related to the lumbar spine. Furthermore, patients with a back surgery in the year before and after the index imaging were excluded.

A full list of exclusion codes was provided in Exclusion codes, Supplementary Information. For the analysis we included all patients with an imaging in the year 2016 or 2017 (index years). For each included patient, data one year before and two years after the index year, 4 years in total, were provided by GM. Accordingly, the data evaluated were from 2015 to 2019. The long observation period was chosen to reliably identify and exclude patients without non-specific LBP and to map the costs over a longer period. If patients had images in both index years, we grouped the patients according to the imaging in the first index year and reported baseline characteristics based on the first year of index imaging only.

In Switzerland, all citizens have a compulsory health insurance that covers medical treatments and thus there is no distinction between a public and private system. Insured persons had a deductible for their health care insurance. We defined three levels of deductible amounts: level 1: ≤ 500 Swiss Francs (CHF), level 2: 501–1500 CHF, and level 3: 1501–2500 CHF. Average price 2019: 1 Swiss Franc ≈ 0.90 Euro [14]. In addition, two main models of health care insurances, which could predict health care costs were: managed care insurance and free choice model. In the first one, a healthcare manager (a family doctor [family physician model], a network [network model] or a specialized telemedicine call-centre [telemedicine model]) acts as a gatekeeper. In the second one, insurers have full freedom to receive treatment from doctors and specialists of their choice.

Pharmaceutical cost groups (PCG) were used as a surrogate marker for existing comorbidities. The PCG system is based on the linkage between medication, defined by Anatomical Therapeutic Chemical Classification System (ATC) codes, and specific diagnosis (for example, the use of insulin as a surrogate for diabetes mellitus) and it is used to identify patients with cost-intensive chronic diseases [15]. Moreover, as a possible factor influencing costs, we considered the language spoken in the area of patient residence. In Switzerland, in fact there are three official languages at national level: German, French, and Italian. Some Swiss cantons are bilingual and in these cases we considered the language spoken by the majority of the population. To account also for patients whose residence was abroad, we grouped the area of residence into four categories: German, French, Italian and abroad.

### Health care costs (outcomes)

The overall costs for LBP included all invoices that were reimbursed within the framework of compulsory health insurance, including the cost-sharing of the insured persons. TARMED tariff values were calculated by dividing claims in Swiss Francs by a reimbursement factor for medical services, negotiated annually between medical associations, health insurers, and health authorities on a cantonal level.

In addition to the overall costs, costs for pain medications, LBP-associated additional co-medications and medical services were analysed. Pain medications and LBP-associated additional co-medications (muscle relaxants, proton pump inhibitors, laxatives, sleeping pills, and antidepressants) were identified using the ATC codes. All used ATC codes, within the ATC code list, for (i) pain medication and (ii) other medications were provided as Supplementary Information. The annual average number of prescribed items per patients was also reported together with the costs of pain and additional medication. Medical services included all settled costs in the context of health care utilization, excluding physicians and hospitals, i.e.: occupational therapy, physiotherapy, care services, rehabilitation, assist devices, or complementary medicine. The annual average number of medical service consultations per patient was reported.

To determine incremental costs for patients with LBP, we analysed mean health care costs for patients with LBP (separately for both index years) and compared the costs with a one-to-one matched random sample of the general population without LBP, insured by GM. All patients were at least matched by age and sex. In addition, 60,699 (80.6%) patients could be matched by the following additional variables: canton of residence, type of health care insurance, deductible and all 34 different comorbidities listed in supplemental Table S2 (full matches). If a full match was not possible, we matched patients by all above-mentioned variables and presence of comorbidities overall (dichotomous variable yes/no) (11,810 patients, 15.7%). The remaining patients were matched by age, sex, type of insurance, and presence of comorbidities overall (*n* = 2779, 3.7%), or age, sex, and presence of comorbidities overall (*n* = 8, < 0.1%).

All costs were described and reported annually, in particular for the index year, and as average of the 4-year period. Costs were reported split by index year cohort or combined. In the latter case, the sum or the average by index year, was considered, respectively, for total costs and patient level costs. Since data were collected from different years (2015 to 2019), all costs were adjusted to the same reference year (2019) using consumer price index to let costs comparable. Consumer Price Index (CPI) for Switzerland in 2015–2019 were taken from [16] and reported in Consumer Price Index and Inflation rates (2015–2019), Supplemental Information. Costs were expressed in Swiss Francs but to let costs comparable with the ones in the European context, we converted and reported the main results into Euro (EUR) too, adjusting also for price levels indices for health care services [17] in Switzerland in 2019 with reference to the European Union average price level. Comparative price levels for health sector were taken from Eurostat [18]. CPI, inflation rates and adjusting factors are reported in the Supplemental Information, Table S3.

### Statistics

Descriptive statistics of basic characteristics of the patients were reported as number (percentage %) or means (standard deviation [SD]) where appropriate. Group comparisons by diagnostic image and by index year were performed using Chi-square tests, for categorical variables, or ANOVA test, for continuous variables (for example age). Patient characteristics considered were: age, sex, survival status, language spoken in canton of residence, health insurance model and relative deductible, comorbidities, use of medical service and in-hospital treatment. Mortality rate of LBP patients was compared with the mortality rates of the general population, whose calculation was based on [19, 20]. Health care costs, total and per patient, specific for index year only or as average of the four year period, were presented overall, by index year and diagnostic imaging. Costs group comparisons were performed through ANOVA tests. In order to account for the skewed data, bootstrap resampling method, case bootstrap, was used to compute p-values.

A multivariable mixed model with patients as random effects (intercept) and correction for autocorrelation, autoregressive moving average ARMA (1,1), was implemented to study the association of patient variables with the average yearly cost per patient. Details of the model were provided in Statistical model description, Supplementary Information. Patient characteristics, described above, were considered into the model together with the index imaging year (2016 or 2017) and the time from index imaging. Effect of time, from index imaging, was not linear on costs. Therefore, a quadratic trend was used. We also considered in the model, as fixed effect, the interaction term between the type of insurance and the deductible. To address skewed data, results of the regression were estimates and confidence intervals computed using bootstrap resampling method for mixed models (case bootstrap with resampling at patient level) [21].

For group comparisons of costs and for the regression analysis, patients with images in both index years were excluded to avoid overestimation. However, as additional analysis, for these patients the trend of annual costs, during all the study period, was showed graphically by type of index imaging in both index years.

Annual costs for LBP patients compared with annual costs for no LBP patients depending on age were showed graphically for all patients matched at least by age and sex. A regression analysis of annual costs of the two subgroups, with full matches including comorbidities, is provided as Supplemental Information, Table S4. In the latter analysis, patients with two or more comorbidities were excluded because information on the costs split by comorbidities was missing. Results of all regression analyses were reported as estimates and 95% confidence intervals.

For all tests, *P* ≤ 0.05 was considered statistically significant. All analyses were carried out using statistical package R (https://www.R-project.org).

### Ethics

Analysing full anonymized and aggregated data from health care insurances did not fall under the Swiss Federal Act on Research involving Human Beings (Human Research Act) and thus no ethics approval was needed.

## Results

### Basic characteristics

**Fig. 1:**
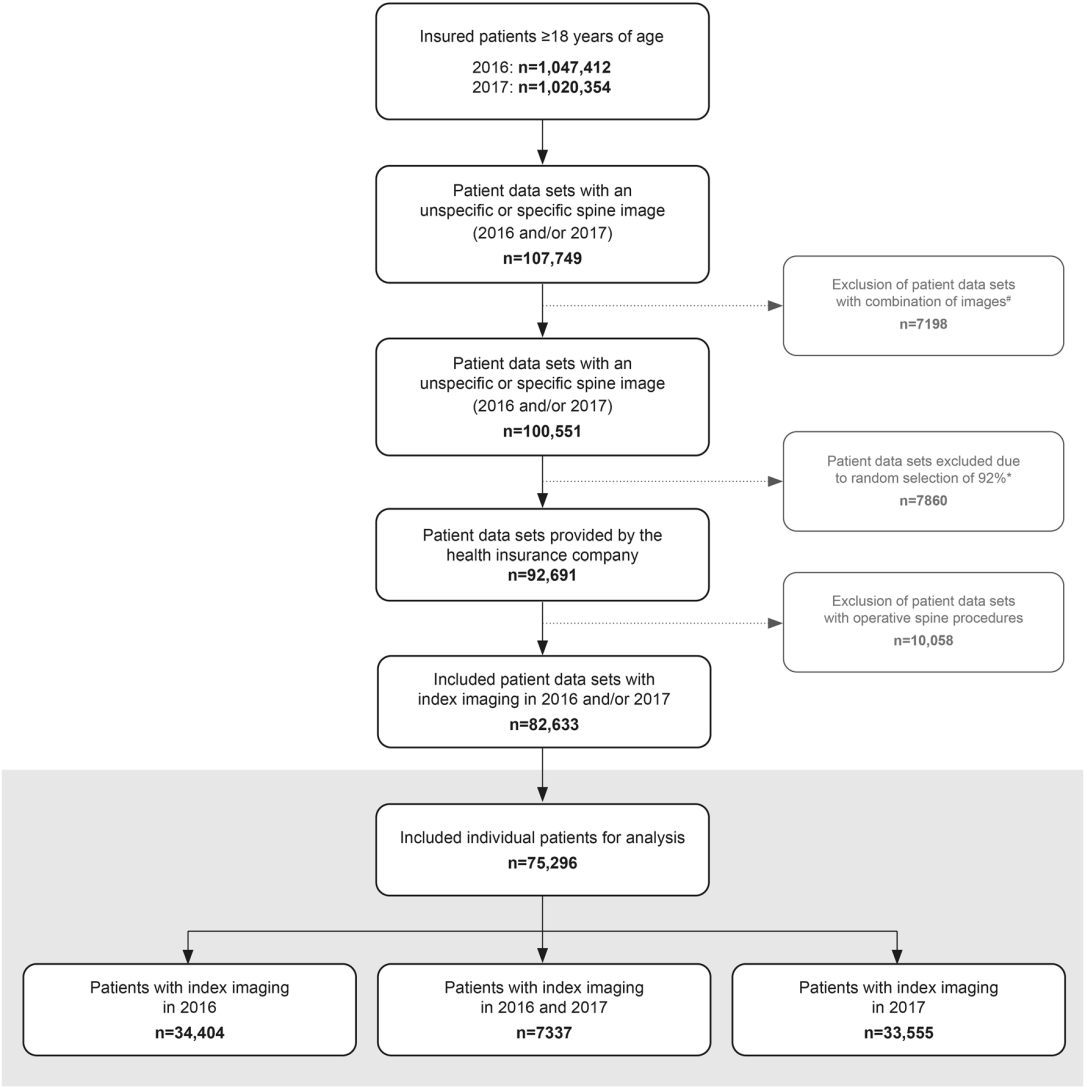
Flowchart of 75,296 eligible patients with an index imaging in 2016 and / or 2017. Patient exclusions are described in the boxes to the right: ^#^patients with an unspecific radiological image (computed tomography [CT] or magnetic resonance imaging) of the spine and a simultaneous radiological image of an adjacent anatomical region were excluded (for example CT of the spine and CT of the shoulder). *Due to applicable regulations provided by the health care insurance, a random selected subset of around 92% of all identified patients could be analysed. Grey shaded area: detailed description of individual patients (versus patient data sets in which patients may be listed twice, if index imaging was in 2016 and 2017)

We included 75,296 patients in our analysis (Fig. 1) for both index years combined. Patients with any imaging of the spine corresponded to 4.4% of all insured patients in the index year. The mean age was 54.5 years (SD 16.4 years) and 57% were female.

Most of the patients were from the French (56%), and German-speaking part (39.4%) of Switzerland. A proportion of 17.5% had an in-hospital treatment during the observation period. An MRI of the spine was the most common imaging method (44.3%), followed by X-ray (32.5%) and a combination of X-ray/CT imaging (11.3%). We found statistically significant differences in all analysed variables in dependence of the type of index imaging (*p* < 0.001 for all variables). Patients with a CT imaging were older and had more comorbidities compared to patients with an X-ray or MRI imaging. In addition, both the proportion of deceased and the mortality rate compared to the general population were highest in patients with a CT (Table 1 and Supplementary Information Table S1). A detailed table of comorbidity frequencies overall and by diagnostic imaging is provided in Table S2, Supplementary Information.

**Table 1:** Basic characteristics of 75,296 patients according to the index year and the type of diagnostic image in the index year

	Total	Index year		Type of diagnostic image					
	N = 75,296	2016 41,741 (55.4)	2017 33,555 (44.6)	X-ray 24,503 (32.5)	CT^a^7,599 (10.1)	MRI 33,393 (44.3)	X-ray + CT 1,319 (1.7)	X-ray + MRI 8,482 (11.3)	
Age, mean (SD)	54.48 (16.42)	54.58 (16.39)	54.37 (16.47)	55.58 (17.92)	60.02 (15.56)	52.05 (15.05)	62.88 (15.76)	54.65 (15.94)	
Sex									
F	42,884 (57.0)	23,880 (57.2)	19,004 (56.6)	14,667 (59.9)	3,920 (51.6)	18,834 (56.4)	786 (59.6)	4,677 (55.1)	
M	32,412 (43.0)	17,861 (42.8)	14,551 (43.4)	9,836 (40.1)	3,679 (48.4)	14,559 (43.6)	533 (40.4)	3,805 (44.9)	
Deceased	3,096 (4.1)	1973 (4.7)	1123 (3.3)	1,127 (4.6)	750 (9.9)	847 (2.5)	116 (8.8)	256 (3.0)	
Area of residence by language	missing = 6	missing = 3	missing = 3		missing = 1	missing = 3		missing = 2	
German	29,643 (39.4)	16,234 (38.9)	13,409 (40.0)	8,987 (36.7)	2,425 (31.9)	13,941 (41.8)	370 (28.1)	3,920 (46.2)	
French	42,198 (56.0)	23,600 (56.5)	18,598 (55.4)	14,503 (59.2)	4,928 (64.9)	17,709 (53.0)	877 (66.5)	4,181 (49.3)	
Italian	3,091 (4.1)	1717 (4.1)	1374 (4.1)	926 (3.8)	187 (2.5)	1568 (4.7)	62 (4.7)	348 (4.1)	
Abroad	358 (0.5)	187 (0.4)	171 (0.5)	87 (0.4)	58 (0.8)	172 (0.5)	10 (0.8)	31 (0.4)	
Health insurance model									
Free choice model	31,324 (41.6)	18,052 (43.2)	13,272 (39.6)	10,399 (42.4)	3,727 (49.0)	12,983 (38.9)	699 (53.0)	3,516 (41.5)	
Family Physician	19,385 (25.7)	10,415 (25.0)	8970 (26.7)	6,387 (26.1)	1,578 (20.8)	8,901 (26.7)	281 (21.3)	2,238 (26.4)	
Model telemedicine	12,959 (17.2)	6790 (16.3)	6169 (18.4)	4,083 (16.7)	1,119 (14.7)	6,161 (18.4)	180 (13.6)	1,416 (16.7)	
Network model	11,628 (15.4)	6484 (15.5)	5144 (15.3)	3,634 (14.8)	1,175 (15.5)	5,348 (16.0)	159 (12.1)	1,312 (15.5)	
Deductible^b^									
Level 1	59,893 (79.5)	33,561 (80.4)	26,332 (78.5)	19,642 (80.2)	6,164 (81.1)	26,093 (78.1)	1,152 (87.3)	6,842 (80.7)	
Level 2	9,271 (12.3)	5104 (12.2)	4167 (12.4)	2,852 (11.6)	910 (12.0)	4,394 (13.2)	112 (8.5)	1,003 (11.8)	
Level 3	6132 (8.1)	3076 (7.4)	3056 (9.1)	2,009 (8.2)	525 (6.9)	2,906 (8.7)	55 (4.2)	637 (7.5)	
Use of medical services									
Yes	71,863 (95.4)	39,968 (95.8)	31,895 (95.1)	22,647 (92.4)	7,131 (93.8)	32,468 (97.2)	1,296 (98.3)	8,321 (98.1)	
No	3,433 (4.6)	1773 (4.2)	1660 (4.9)	1,856 (7.6)	468 (6.2)	925 (2.8)	23 (1.7)	161 (1.9)	
Comorbidities									
Yes	24,741 (32.9)	14,174 (34.0)	10,567 (31.5)	7,803 (31.8)	3098 (40.8)	10,285 (30.8)	636 (48.2)	2,919 (34.4)	
No	50,555 (67.1)	27,567 (66.0)	22,988 (68.5)	16,700 (68.2)	4501 (59.2)	23,108 (69.2)	683 (51.8)	5,563 (65.6)	
In-hospital treatment	Missing = 159	Missing = 80	Missing = 79	Missing = 58	Missing = 13	Missing = 64		Missing = 24	
Yes	13,140 (17.5)	7366 (17.7)	5774 (17.2)	3,928 (16.1)	2,171 (28.6)	5,237 (15.7)	357 (27.1)	1,447 (17.1)	
No	61,997 (82.5)	34,295 (82.3)	27,702 (82.8)	20,517 (83.9)	5415 (71.4)	28,092 (84.3)	962 (72.9)	7011 (82.9)	

If not stated else, variables are presented as absolute numbers and percentages (in parentheses)

^a^*CT* computed tomography, *MRI* magnetic resonance imaging, *SD* standard deviation, *N* number of patients, *F* female, *M* male

^b^Deductible levels: level 1: ≤ 500 Swiss francs (CHF), level 2: 501–1500 CHF, and level 3: 1501–2500 CH

Regarding year of index imaging, in 2017, compared to 2016, we found significantly higher proportion of patients in the German area (*p * = 0.012), significantly higher proportion of telemedicine insurance model and highest franchise (*p *< 0.001). In contrast, we found significantly lower proportion of comorbidities, deceased and use of medical services (*p * < 0.001).

### Health care costs

Main health care costs are presented in in Table 2 (in CHF) and in Supplemental Information Table S3 (in EUR). The sum of the annual health care costs (averaged on the whole observation period of 4 years and for the two groups based on index imaging 2016 and 2017) was around 518 million CHF (467 million EUR), 628 million CHF (565 million EUR) including patients with two index imaging in 2016 and 2017. Overall costs for patients, relative only to the year of index imaging, were 319,590,507 CHF (287,631,456 EUR); ambulatory: 266,310,563 CHF (239,679,507 EUR), in-hospital: 53,279,944 CHF (47,951,950 EUR) for group 2016, n = 34,404 patients, and 320,910,867 CHF (288,819,780 EUR); ambulatory: 268,191,856 CHF (241,372,670 EUR), in-hospital: 52,719,011 CHF (47,447,110 EUR) for group 2017, *n* = 33,555 patients.

**Table 2:** Annual health care costs (in Swiss Francs, CHF) for all patients with only one index imaging

	Total/overall	X-ray	CT	MRI	X-ray and CT	X-ray and MRI
Number of patients^a^	67,959	22,474	6537	30,392	1062	7494
Cohort 2016	34,404	11,579	3150	15,274	536	3865
Cohort 2017	33,555	10,895	3387	15,118	526	3629
Overall annual costs^b^	518,488,470	158,572,216	70,727,315	218,641,017	12,583,619	57,964,303
Total: cohort 2016^c^	258,863,732	80,942,939	32,608,029	110,145,440	6,381,625	28,785,698
Total: cohort 2017^c^	259,624,738	77,629,276	38,119,286	108,495,576	6,201,994	29,178,605
Ambulatory: cohort 2016	212,603,762	65,020,098	25,649,547	93,068,012	5,116,691	23,749,414
Ambulatory: cohort 2017	213,846,043	63,203,610	30,347,419	91,357,092	4,766,658	24,171,264
In-hospital: cohort 2016	46,259,970	15,922,841	6,958,483	17,077,429	1,264,933	5,036,285
In-hospital: cohort 2017	45,778,695	14,425,666	7,771,867	17,138,484	1,435,336	5,007,341
Individual: cohort 2016^d^	8571	8008	11,590	8214	13,178	8496
Individual: cohort 2017^d^	8878	8223	12,687	8256	13,105	9169
Individual subgroup: cohort 2016^e^	22,849	23,144	24.373	22,175	26,523	21,325
Individual subgroup: cohort 2017^e^	23,447	22,305	27,321	22,936	24,791	21,770
Individual index year cost: 2016^f^	9289	7977	14,091	9031	14,324	9632
Individual index year cost: 2017^f^	9564	8113	14,906	9157	14,976	9844
Costs for imaging^g^	18,180,182	639,463	1,778,577	12,191,754	315,636	3,254,751
Individual index year cost: 2016	261	28	270	394	297	420
Individual index year cost: 2017	274	29	274	409	298	450
Pain medication costs	5,245,791	1,480,829	596,017	2,305,477	147,592	715,876
Individual: cohort 2016	126	110	160	122	201	152
Individual: cohort 2017	122	109	137	122	192	137
Quantity of prescriptions: cohort 2016^h^	6	6	8	6	8	7
Quantity of prescriptions: cohort 2017^h^	6	6	6	6	9	7
Individual index year cost: 2016	124	105	159	120	189	156
Individual index year cost: 2017	122	105	134	122	198	144
Additional medication costs	5,906,696	1,831,683	673,056	2,534,470	139,053	728,434
Individual: cohort 2016	183	182	206	175	221	190
Individual: cohort 2017	180	183	200	171	221	184
Quantity of prescriptions: cohort 2016^h^	5	5	6	5	6	6
Quantity of prescriptions: cohort 2017^h^	5	5	6	5	7	5
Individual index year cost: 2016	167	168	195	159	202	171
Individual index year cost: 2017	167	170	196	157	214	167
Costs for medical services	84,521,978	24,180,027	9,276,552	38,413,941	1,893,809	10,757,649
Individual: cohort 2016	1889	1746	2266	1857	2447	2021
Individual: cohort 2017	1951	1794	2172	1929	2483	2194
Number of consultations: cohort 2016^i^	14	12	13	14	19	15
Number of consultations: cohort 2017^i^	14	12	13	14	15	15
Individual index year cost: 2016	2007	1584	2506	2099	2622	2300
Individual index year cost: 2017	2061	1623	2340	2151	2774	2505

Costs are observed during 2015–2019 and split by the index year cohort and the type of diagnostic imaging

Values are rounded to the nearest integer and corrected for inflation rates expressing all costs in 2019 prices in Switzerland (details in Supplemental Information)

^a^The number of patients is defined at the index year. However, for not all patients we have 4 years of observations

^b^Average annual cost, summed by cohort. The total costs are the sum of ambulatory and in-hospital treatment costs

^c^Average annual total cost split by patients with only one index imaging in 2016 (cohort 2016) or in 2017 (cohort 2017)

^d^Average annual total cost per patient, split by patients in cohort 2016 or cohort 2017

^e^Average annual total cost per patient, split by index year cohort 2016/2017 and relative to the subgroup of patients with comorbidities and in-hospital treatment

^f^Average cost per patient relative to the index year only, split by cohort 2016/2017

^g^Costs for imaging studies are only reported for the index year

^h^Annual average quantity of prescribed items per patients, split by index year cohort 2016/2017

^i^Annual average number of consultations per patients, split by index year cohort 2016/2017

The mean annual health insurance costs per patient, regardless of the type of index imaging and index year was 8722 CHF (7850 EUR) for the whole observation period. Annual costs for patients with only one index imaging, in relation to the index year and the type of diagnostic imaging are presented in CHF, Table 2. The main results converted in EUR, adjusted for the European Union average price level indices in health sector in 2019, are shown in Supplemental Table S3. Lowest mean costs per patient were observed in patients with an X-ray as index imaging, 8112 CHF (7301 EUR), and highest in patients with a CT + X-ray, 13,142 CHF (11,828 EUR). This pattern of costs distribution was almost the same for each type of expense in our analysis: patients with only X-ray imaging had the lowest costs, whereas patients with X-ray and additional CT imaging the highest costs.

**Fig. 2:**
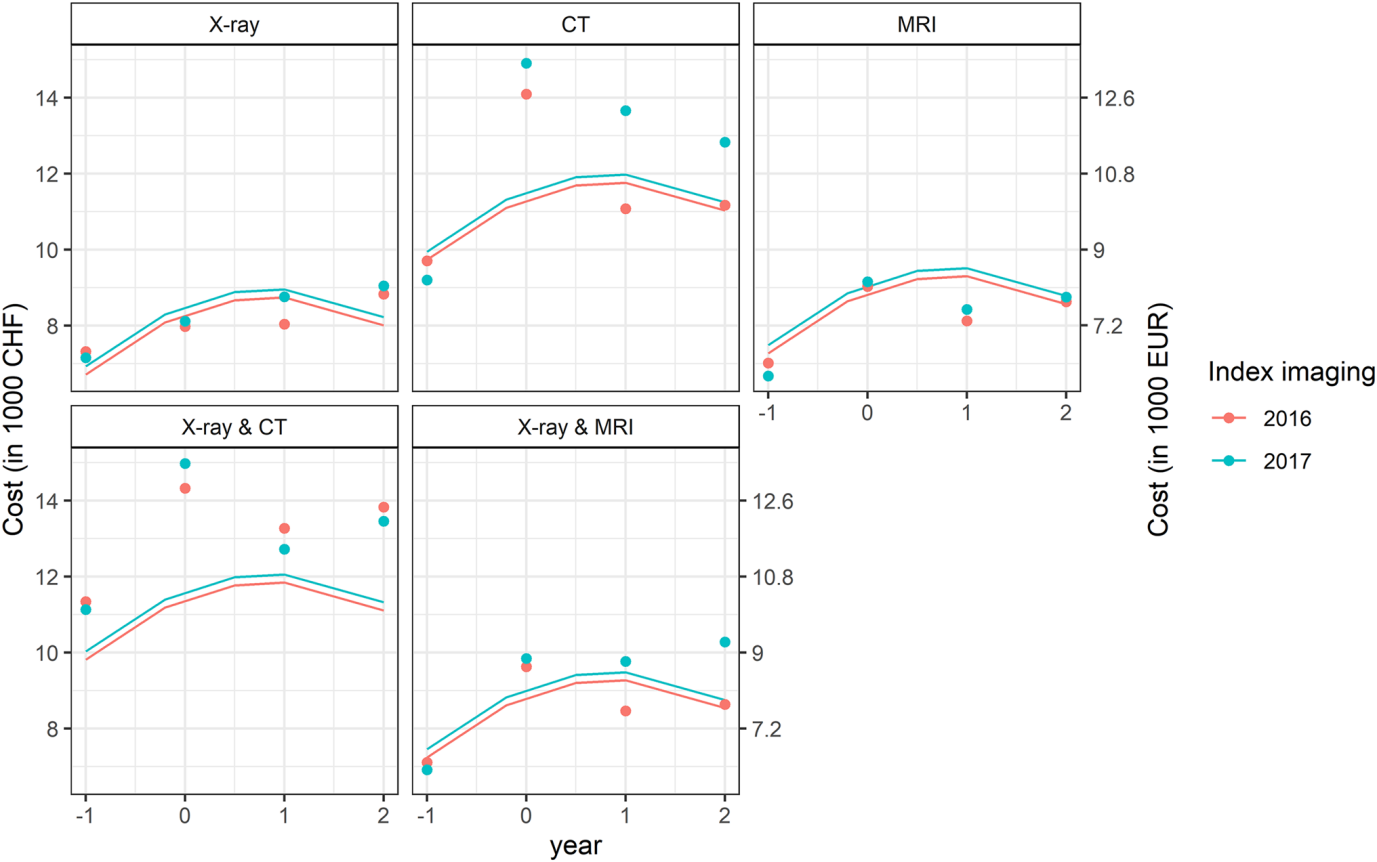
Mean costs per patient depending on the kind of index imaging. Dots represent the mean costs per patient in 1000 Swiss Francs (CHF), left y-axis, and 1000 Euro (EUR), right y-axis. Curves represent the fitted line from the regression models. Red: index imaging year 2016, blue: index year 2017. CT: computed tomography; MRI: magnetic resonance imaging. Year goes from -1 (one year before index imaging year) to 2 (two years after index year). Values are corrected for inflation rates expressing all costs in 2019 prices in Switzerland (details in Supplemental Information)

The individual costs for analgesic drugs, during the index year, ranged from 105.10 CHF (94.59 EUR), for patients with X-ray, to 193.67 CHF (174.30 EUR) for patient with X-ray and CT. The costs for additional drugs in the index year ranged from 157.96 CHF (142.16 EUR), for patients with MRI, to 207.99 CHF (187.19 EUR) for patients with X-ray and CT. Costs for medical services were lowest in the X-ray group and highest in the CT group: 1602.78 CHF (1442.50 EUR) vs 2698.29 CHF (2428.46 EUR) in the index year. Mean costs of all patients over the whole observation period were plotted as points in Fig. 2. Costs for patients with the index imaging in 2017 were slightly higher compared to the costs for patients with the index imaging in 2016. In general, costs were highest in the year of index imaging.

An additional analysis for the 7337 patients with a double index imaging in 2016 and 2017 is reported in Fig S1, Supplementary Information. Over all observation period, in this patient subgroup the costs were higher compared to the ones of the patients with a single index imaging.

### Regression modelling

Patient variables and their association to the overall costs, at patient level, are analysed in Table 3 and Supplementary Information, Table S4. The use of medical services and the presence of comorbidities were associated with the highest increase in costs: 2540 CHF (2286 EUR) and 6178 CHF (5560 EUR), respectively. On the other hand, living outside the French-speaking part of Switzerland and having an insurance model with a gatekeeper function were associated with lower costs. Moreover, the regression results confirmed the significant highest costs (*p * < 0.001) in patients with a CT + X-ray. Fitted values of the model, by diagnostic imaging and index imaging group (2016 vs. 2017) are shown in Fig. 2. Within the comorbidities variable, for patients with only one comorbidity matched with no LBP patients, we found a heterogeneous distribution of costs ranging from 868 CHF (781 EUR) for cardiovascular diseases up to 35,465 CHF (31,918 EUR) for cancer, Supplementary Information, Table S4.

**Table 3:** Regression model analysing patient variables (fixed effects) affecting average cost per patient (in Swiss Francs)

Variable^a^	Estimates	95% CI	*p*
Age	101.68	(92.78, 102.15)	< 0.001
Male gender	– 165.21	( – 259.29, 20.66)	0.066
Medical services	2540.37	(2340.71, 2777.4)	< 0.001
Comorbidity	6178.18	(5876.76, 6262.13)	< 0.001
Area of residence (ref: French speaking)			
Abroad	– 1745.10	( – 2608.81, – 1235.45)	0.005
German speaking	– 863.76	( – 945.23, – 679.13)	< 0.001
Italian speaking	– 1034.81	( – 1350.97, – 729.56)	< 0.001
Type of insurance (ref free choice model)			
Network model	– 944.77	( – 1540, – 573.26)	0.009
Family physician model	– 780.97	( – 1057.51, -255.8)	0.009
Telemedicine model	– 974.19	( – 1299.59, – 485.27)	0.001
Deductible (ref: 2500)			
≤ 500	1794.48	(1910.94, 2646.48)	< 0.001
≤ 1500	– 66.17	( – 294.7, 611.82)	0.815
Year^2^	– 580.93	( – 629.44, – 573.26)	< 0.001
Year	1013.66	(996.7, 1095.78)	< 0.001
Imaging in 2017 (ref 2016)	214.08	(58.85, 349.44)	0.015
Type of imaging (ref X-ray)			
X-ray + CT	3101.69	(2369.36, 3641.61)	< 0.001
X-ray + MRI	530.33	(247.06, 639.81)	0.006
CT	3019.64	(2498.08, 2997.51)	< 0.001
MRI	557.91	(341.41, 591.8)	< 0.001
Interaction (type of insurance and deductible)			
Network model and deductible ≤ 1500	593.90	(-283.79, 886.23)	0.181
Family physician model and deductible ≤ 1500	489.54	( – 518.17, 810.39)	0.216
Telemedicine model and deductible ≤ 1500	431.28	( – 514.49, 795.63)	0.283
Network model and deductible ≤ 500	– 65.34	( – 807.45, 184.78)	0.862
Family physician model and deductible ≤ 500	– 36.89	( – 917.86, 15.61)	0.906
Telemedicine model and deductible ≤ 500	– 359.18	( – 1132.54, – 237.94)	0.263

Estimates and empirical 95% confidence intervals (CI) were computed using case bootstrap with 100 resamples at patient level

^a^Mixed model with patients as random effects and correction for autocorrelation ARMA (1,1). Patients with images in both index years were excluded from the analysis. All estimates are in Swiss Francs. Numbers of observations = 240,982, numbers of patients/insured = 67,954. *Ref* reference, *CT* computed tomography, *MRI* magnetic resonance imaging, time was defined as 0 (index imaging), -1 (the year before index imaging), 1, 2 (first, second year after index imaging). Effect of time on costs was not linear (quadratic trend) with coefficients Year^2^ + Year reported, that means the costs are first increasing then decreasing on year from index imaging

### Estimation of incremental costs

**Fig. 3:**
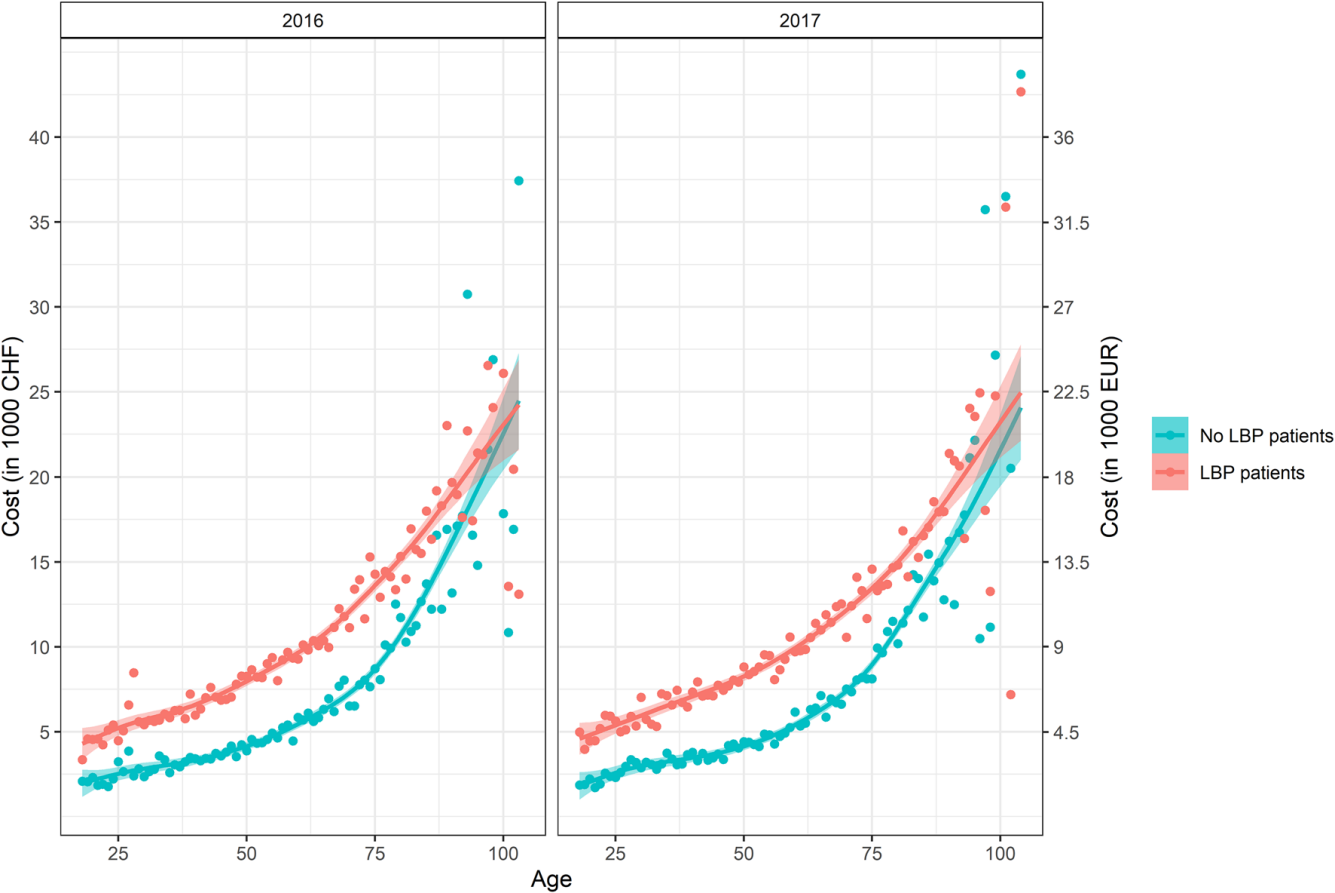
Mean health care costs per patient, in 2016 and 2017 separately, by age (in years), of LBP patients compared to a comparable population, insured by GM, without LBP. For each age, average costs per patient are represented with points and lines represented the smoothed curves. Shaded areas represented the 95% Confidence Interval of the fitted curves. Costs are expressed in 1000 Swiss Francs (CHF), left *y*-axis, and 1000 Euro (EUR), right *y*-axis, and corrected for inflation rates expressing all costs in 2019 prices in Switzerland (details in Supplemental Information)

Health care costs relative to a population of no LBP insured persons, randomly matched by a one-to-one allocation, are shown in Fig. 3. In patients ≤ 90 years of age, the average health care costs for LBP patients were higher than the average costs of no LBP insured persons with an absolute difference ranging from 852 to 7642 CHF (767–6878 EUR), in 2016, and from 1232 to 6467 CHF (1109–5820 EUR), in 2017, and an overall relative difference of 82% of the average costs for no LBP patients. For patients aged above 90 years, the overall difference of 9% was not significant. For all ages, the average relative difference was of 72% of the average costs for no LBP patients. Regarding the comorbidities groups, the following ones had greater estimates of costs for LBP patients compared to no LBP patients: cancer, 35,465 CHF (31,918 EUR) for LBP vs 20,269 CHF (18,242 EUR) for No LBP; neurological diseases 10,507 CHF (9456 EUR) LBP vs 9443 CHF (8499 EUR) no LBP; psychological diseases 4454 CHF (4009 EUR) LBP vs 4274 CHF (3847 EUR) no LBP; other diseases 2833 CHF (2550 EUR) LBP vs. 2330 CHF (2097 EUR) no LBP (Supplementary Information, Table S4).

## Discussion

In this study, we reported patient characteristics and overall health care costs of patients with a radiological imaging for LBP in Switzerland. We found that a large proportion of insured persons had a radiological imaging of the spine and that this group contributes substantially to the health care costs. In our patient group of > 75,000 patients, costs were highest in patients with a CT imaging (alone or combination with X-ray) at patient level whereas, on an insurance level cost, were highest in patients with an MRI or X-ray imaging. LBP patients' costs were higher than those of the general insured at GM, especially the younger the patients were.

### Importance, background, and characteristics

Imaging examinations of the lumbar spine are common in patients with LBP [22]. To improve quality of care and to limit unnecessary health care expenses and associated economic burden, many guidelines recommend imaging studies only if patients have “red flags” or a serious suspected pathology [10]. Although, some evidence suggests that many patients with LBP, seen in primary care, have a “red flag” [23] and thus justifies the high numbers of radiological imaging, there is a substantial proportion of inappropriateness in patient management and many initiatives call for a more prudent approach in patient care [11, 12]. In addition, evidence-based treatment of LBP patients was associated with lower treatment costs [24]. In our analysis, we included patients with a radiological imaging of the spine as a surrogate for LBP. In the literature, the proportion of patients with LBP with a radiological imaging differs [22, 25] as well as the proportion of used imaging techniques, as it depends on the clinical setting. In line with our results, which are based on a sample of 12% of the Swiss population, MRI and X-ray imaging are often the most common used methods [26]. In our cohort, distribution of both age and sex was similar compared to previous reports on LBP in Switzerland [5, 27]. We found that, in our cohort, both the proportion of patients with a deductible ≤ 500 CHF and insurance model without a gatekeeper (free choice of medical practitioner model) were higher compared to the general population [28]. In fact, the proportion in our study were, in the first case 76.5% vs. 56.5% and in the second case 40.3% vs. 29.8%.

### Health care costs

LBP contributes substantially to national health care costs. This is due to the combination of the high prevalence of LBP and the high individual costs for affected patients. In the United States, low back and neck pain account for the third highest health care spending and expenses have been continuously increasing over the last decade [29]. Also in our analysis, index imaging in 2017 were associated with higher costs compared to 2016 and mean health care costs were higher over the entire observation period in patients with an index image in 2017 than in patients with an index image in 2016. At patient level, we found the highest costs for patients with CT imaging (CT alone or in combination with an X-ray). CT is indicated if bone structures are critical [30, 31] and patients in this subgroup had the highest rates of comorbidities, in-hospital treatments, and a higher mortality rate. In addition, at patient level, costs for pain medication, additional medications and also other medical services were highest in this group. In our patient group, a CT scan of the lumbar spine could be seen as a surrogate of a more severe overall health status compared to patients undergoing a MRI scan or a conventional X-ray and thus explaining the higher costs at patient level.

At health insurance level, due to the more frequent use of these imaging methods, costs were highest for patients with an MRI or X-ray examination. In contrast to CT imaging, MRI is preferred for investigations with focus on soft tissue structures like nerve or lumbar disc pathologies [32]. Comorbidities, in-hospital treatments and mortality rates were lower compared to the subgroup with CT imaging and costs for pain medications, additional medications and other medical services were lower too.

For Switzerland, only sparse literature exists. Wieser et al. [8] reported total yearly costs for LBP of about 6.6 Billion euro in 2005, which equals 6.1% of the national health care spending in 2005. Direct medical costs were estimated with 2.5 billion euro, and indirect costs (using a human capital approach) with 4.1 billion euro. This calculation relied on estimates based on self-declarations obtained by a survey among a convenience sample of the general population and reports LBP-associated costs only. However, a direct comparison with our data is not possible due to the different methodologies used and population studied.

In contrast, we were able to include in our study only patients with LBP who had received diagnostic imaging. Our study lacks of all health care costs of patients without an imaging study, thus our numbers underestimate the real economic burden of LBP in Switzerland. Furthermore, it should be considered that many patients bear LBP-associated costs themselves and thus these costs could not be included in our analysis [5]. With reference to the population insured by GM, an age-adjusted comparison of mean costs shows that patients with LBP have higher costs than the average insured person as long as they are younger than 90 years of age. The costs surpass the mean health care costs by 72%, on average, and were higher the younger the patients were. Our findings could confirm findings in the literature, reporting that the mean costs for LBP patients surpass the costs of the general population [4, 33]. Optimized preventive and therapeutic efforts should therefore, from a purely economic point of view, focus primarily on younger patient groups, where the greatest difference in health care costs compared to the general population can be observed. With increasing age, the prevalence of chronic diseases increases and there is a growing possibility that the presence of LBP has a potentiating interaction with concomitant diseases. Differentiated analyses are therefore needed to analyse the effect of LBP on health care costs as patients get older.

### Characteristics associated with costs

Identifying patients with an increased risk of high costs is of utmost importance. By identifying the population at risk and specific subgroups of even higher risk, not only health care insurance companies, but all relevant parties in a health care system could improve allocation of efforts or could apply preventive measures in advance. We identified specific patient characteristics affecting patient health care costs. For some characteristics, for example comorbidities and use of other health care utilization, an explicit association with increased costs is understood. In addition, we could show that the area of residence, type of insurance and the level of deductibles were associated with increased or decreased costs. These findings are in line with evidence suggesting that low deductibles and an insurance model without any gatekeeper function are associated with higher costs, since managed care models in Switzerland seem to be more economic [34, 35]. With regard to comorbidities, we found a varying influence of the different comorbidities on health care costs. At the same time, this influence was partly even greater in the matched cohort of non-LBP patients. Further studies are therefore needed to investigate the complex interplay between comorbidities and health care costs in patients with LBP.

### Strengths and limitations

In this study, we reported actual data on patient in a large group of > 75,000 patients, provided by one of the biggest health care insurance companies in Switzerland. The knowledge of the distribution of used radiological imaging studies, patient characteristics and health care costs in this population, will improve strategies and future interventions fostering prudent patient care. In accordance with a recently published proposed guidance in reporting costs in patients with LBP [36], our study reported detailed descriptions of used methods and considered costs. We reported detailed patient characteristics including 34 different and well-defined comorbidities.

This study has some limitations. First, due to applicable regulations of GM, we could only analyse a random selected subset of 92% of all patients with LBP. However, due to the random selection of patients and the still high percentage of analysed cases (> 90%) the analysis is still representative for the population insured by GM. In addition, we are aware that GM insured only around 12% of the Swiss population and thus our data might not be representative for all insured people in Switzerland. Second, we could not totally exclude the chance, that patients with unspecific imaging codes had an imaging examination other than or exclusively for the lumbar spine. Third, we could only report the overall medical costs reimbursed by the health care insurer, therefore, we were unable to provide a full breakdown of all costs specific for LBP only. Finally, some aspects and findings of this study are limited to the specific characteristics of the Swiss Health system and the validity of these specific findings might not be extended to other countries.

## Conclusion

In this study, we reported characteristics and overall health care costs for patients with a diagnostic imaging for LBP. Diagnostic imaging for LBP is common and costs for patients with LBP clearly surpassed the average health care costs of insured patients without LBP. Our findings confirm the economic burden of LBP and highlight the importance of ongoing efforts to improve prevention, diagnostic and patient care in patients with LBP.

## Supplementary Information

Below is the link to the electronic supplementary material.

Supplementary file1 (PDF 541 KB)

## Data Availability

Data are available on request due to privacy or other restrictions.

## Code availability

Code for data cleaning and analysis is available on requests.
