# Supplementary material for: Characteristics and health care costs in patients with a diagnostic imaging for low back pain in Switzerland
Source: Eur J Health Econ. 2021 Oct 30;23(5):823–35. doi: 10.1007/s10198-021-01397-8 (PMC9170616; doi:10.1007/s10198-021-01397-8)
Supplement: Supplementary file 1 — Supplementary file1 (PDF 541 KB) [file 10198_2021_1397_MOESM1_ESM.pdf]

Figure S1: Mean health care costs (in Swiss Francs, CHF) in patients with two index images (*n*=7337) depending on the kind of index imaging. Dots represent the mean costs in Swiss Francs (CHF). Curves represent the fitted line from the regression models. Green shaped box: Type of index imaging in 2016, light-pink shaped box: type of index imaging in 2017. CT: computed tomography; MRI: Magnetic resonance imaging.

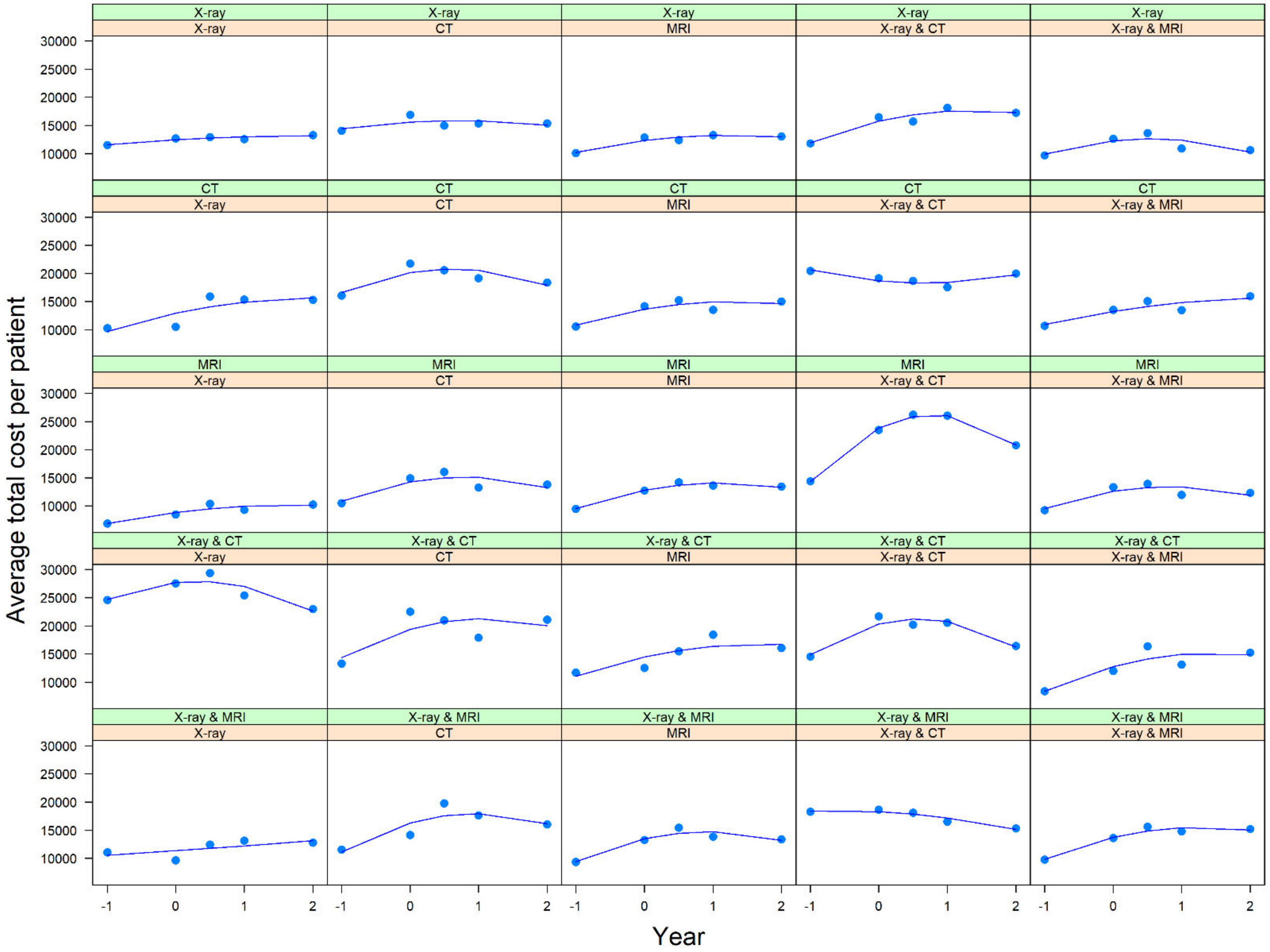

Table S1: Mortality rates.

	Mortality Rate (Population aged ≥18)		By image				
	General Population	Our cohort (excluding patients with two index imaging)	X-ray	CT	MRI	X-ray+CT	X-ray+MRI
	(rate * 1000 inhabitants)	(rate * 1000 patients)					
2016	9.340	9.069	9.068	27.302	5.827	20.522	5.433
2017	9.555	10.021	10.857	25.547	6.120	29.190	7.072

Table S2: Comorbidities by diagnostic image in the index year. Variables are presented as absolute numbers and percentages (in parentheses).

Comorbidity	Overall 75,296	X-ray 24,503	CT 7599	MRI 33,393	X-ray & CT 1319	X-ray & MRI 8482	p
Addiction without nicotine	69 (0.1)	24 (0.1)	11 (0.2)	22 (0.1)	1 (0.1)	11 (0.1)	0.191
ADHD	82 (0.1)	28 (0.1)	8 (0.1)	34 (0.1)	2 (0.2)	10 (0.1)	0.975
Autoimmune disorders	540 (0.8)	158 (0.7)	44 (0.6)	258 (0.9)	4 (0.3)	76 (1.0)	0.014
Alzheimer	101 (0.1)	63 (0.3)	13 (0.2)	18 (0.1)	1 (0.1)	6 (0.1)	<0.001
Asthma	1114 (1.6)	356 (1.6)	173 (2.5)	422 (1.4)	38 (3.1)	125 (1.6)	<0.001
Bipolar disorders	91 (0.1)	38 (0.2)	6 (0.1)	36 (0.1)	2 (0.2)	9 (0.1)	0.365
Cardiac disease	669 (1.0)	267 (1.2)	126 (1.8)	180 (0.6)	32 (2.6)	64 (0.8)	<0.001
COPD / severe Asthma	939 (1.4)	313 (1.4)	193 (2.8)	313 (1.0)	30 (2.5)	90 (1.2)	<0.001
Depression	6990 (10.3)	2208 (10.0)	716 (10.3)	3064 (10.2)	191 (15.6)	811 (10.6)	<0.001
Diabetes Typ-I	943 (1.4)	318 (1.4)	119 (1.7)	347 (1.2)	30 (2.5)	129 (1.7)	<0.001
Diabetes Typ-II	872 (1.3)	273 (1.2)	107 (1.5)	380 (1.3)	19 (1.6)	93 (1.2)	0.276
Diabetes with hypertension	1590 (2.3)	536 (2.4)	211 (3.0)	582 (1.9)	45 (3.7)	216 (2.8)	<0.001
Epilepsy	577 (0.8)	227 (1.0)	69 (1.0)	196 (0.7)	25 (2.0)	60 (0.8)	<0.001
Glaucoma	1593 (2.3)	599 (2.7)	244 (3.5)	542 (1.8)	51 (4.2)	157 (2.0)	<0.001
High cholesterol	7335 (10.8)	2457 (11.2)	924 (13.3)	2904 (9.7)	220 (18.0)	830 (10.8)	<0.001
HIV / AIDS	201 (0.3)	63 (0.3)	24 (0.3)	87 (0.3)	4 (0.3)	23 (0.3)	0.945
Hormone-sensitive Cancers	422 (0.6)	126 (0.6)	83 (1.2)	162 (0.5)	8 (0.7)	43 (0.6)	<0.001
Cancer	18 (0.0)	3 (0.0)	5 (0.1)	9 (0.0)	0 (0.0)	1 (0.0)	0.099
Cancer complex	1095 (1.6)	169 (0.8)	383 (5.5)	428 (1.4)	34 (2.8)	81 (1.1)	<0.001
Crohn's disease/ Ulcerative colitis	125 (0.2)	28 (0.1)	23 (0.3)	55 (0.2)	1 (0.1)	18 (0.2)	0.008
Multiple Sclerosis	188 (0.3)	10 (0.0)	10 (0.1)	164 (0.5)	1 (0.1)	3 (0.0)	<0.001
Kidney diseases	92 (0.1)	22 (0.1)	21 (0.3)	37 (0.1)	1 (0.1)	11 (0.1)	0.002
Pulmonary (arterial) Hypertension	11 (0.0)	6 (0.0)	0 (0.0)	3 (0.0)	1 (0.1)	1 (0.0)	0.147
Parkinson's disease	223 (0.3)	68 (0.3)	28 (0.4)	85 (0.3)	12 (1.0)	30 (0.4)	<0.001
Psoriasis	131 (0.2)	43 (0.2)	12 (0.2)	62 (0.2)	2 (0.2)	12 (0.2)	0.909
Psychosis	436 (0.6)	189 (0.9)	55 (0.8)	136 (0.5)	16 (1.3)	40 (0.5)	<0.001
Rheumatism	340 (0.5)	123 (0.6)	22 (0.3)	143 (0.5)	7 (0.6)	45 (0.6)	0.099
Chronic pain without opioid use	4296 (6.3)	1042 (4.7)	381 (5.5)	2068 (6.9)	110 (9.0)	695 (9.1)	<0.001
Neuropathic pain	549 (0.8)	126 (0.6)	62 (0.9)	269 (0.9)	20 (1.6)	72 (0.9)	<0.001
Thyroid diseases	2247 (3.3)	758 (3.4)	243 (3.5)	921 (3.1)	59 (4.8)	266 (3.5)	0.002
Transplantation	151 (0.2)	70 (0.3)	12 (0.2)	53 (0.2)	3 (0.2)	13 (0.2)	0.008
Growth Disturbance	3 (0.0)	3 (0.0)	0 (0.0)	0 (0.0)	0 (0.0)	0 (0.0)	0.181
Cystic fibrosis / Pancreatic enzymes	109 (0.2)	38 (0.2)	28 (0.4)	27 (0.1)	4 (0.3)	12 (0.2)	<0.001
Central nerve system diseases (excluding Multiple Sclerosis)	279 (0.4)	76 (0.3)	19 (0.3)	142 (0.5)	6 (0.5)	36 (0.5)	0.059

Table S3. Annual health care costs converted in Euro (EUR) and annual health care costs adjusted for Eurostat comparative price level in health sector in 2019 (comparative EU); average price in 2019: 1 CHF / 0.90 EUR; price level indices in 2019 Switzerland / European Union (EU) = 218.2/100.

	Cost in EUR	Comparative EU
Overall annual cost[a]	466,639,621	237,620,747
Individual annual cost[b]	7850	3997
Index year costs[c]	576,451,237	293,538,668
Individual index year cost[d]	8482	4319
Pain medication costs	4,721,212	2,404,121
Individual pain medication cost at index year	111	56
Additional medication costs	5,316.026	2.707,010
Individual additional medication cost at index year	151	77
Costs for medical services	76,069.780	38,736,012
Individual cost for medical services at index year	1830	932

[a] Average annual total cost, summed by index year. The total costs are the sum of ambulatory and in-hospital treatment costs.
[b] Average annual cost per patient.
[c] Average total cost relative to the index year, summed by index year.
[d] Average cost per patient relative to the index year.

Table S4: Subgroup analysis of annual average costs, at patient level and at index year, for Low Back Pain and matched no Low Back Pain patients. Age, sex, comorbidities groups, type of insurance model, deductible and year (cohort 2016 vs cohort 2017) were predictors. One to one matched patients, at year of index imaging, were considered, n=55,470. Estimates and empirical 95% confidence intervals (CI) were computed using case bootstrap with 100 resamples at patient level. Values were expressed in Swiss Francs and corrected for inflation rates (prices at 2019 as reference).

Variable[a]	Low Back Pain patients	No Low Back Pain Patients
Age	120.95 (115.18, 129.16)***	105.77 (99.46, 110.75)***
Male gender (ref female)	-171.25 (-344.01, 24.62)	-236.19 (-385.65, -84.09)**
Comorbidities (ref No comorbidities)		
Autoimmune disorders	17,917.58 (14396.43, 21693.34)***	21,512.87 (16498.37, 25932.9)***
Cancer	35,464.56 (29250.88, 41171.85)***	20,269.15 (15981.58, 24623.65)***
Cardiovascular diseases	867.61 (404.01, 1313.26)***	1181.26 (697.41, 1547.9)***
Endocrine diseases	2266.36 (1543.77, 2848.19)***	2826.43 (2181.54, 3442.74)***
Neurological diseases	10,506.7 (5423.2, 15870.53)***	9443.32 (6266.24, 12706.67)***
Pain syndromes	1550.19 (722.05, 2266.99)***	3307.15 (2347.46, 4112.35)***
Psychiatric diseases	4454.06 (4032.08, 4948.17)***	4274.03 (3714.18, 4728.88)***
Pulmonic diseases	3448.44 (1428.62, 4908.22)***	5369.94 (3691, 7188.71)***
Other diseases	2833.43 (1403.9, 4108.35)***	2330.49 (756.78, 3619.62)***
Type of insurance (ref free choice model)		
Network model	-1482.61 (-1720.53, -1199.34)***	-1141.61 (-1316.8, -962.22)***
Family Physician model	-1125.61 (-1355.36, -883.89)***	-800.27 (-987.31, -606.01)***
Telemedicine model	-1386.36 (-1629.53, -1153.38)***	-905.27 (-1100.61, -690.36)***
Deductible (ref: 2500)		
≤ 500	1903.42 (1642.23, 2076.72)***	1990.23 (1815.74, 2161.17)***
≤ 1500	295.72 (45.13, 576.02)	199.59 (-15.13, 423.88)
Year = 2017 (ref 2016)	139.69 (-26.82, 348.64)	40.37 (-101.54, 175.15)

[a] Linear model. Patients with images in both index years and patients with two or more comorbidities were excluded from the analysis. Reference for diseases: no comorbidities. *:p<0.05; **: p<0.01; ***: p<0.001

Pharmaceutical cost groups (PCG) list: diagnosis and comorbidity group

Code	Diagnosis	Comorbidity Group
ABH	Addiction without nicotine	Psychiatric diseases
ADH	ADHD	Psychiatric diseases
AIK	Autoimmune disorders	Autoimmune diseases
ALZ	Alzheimer	Neurological diseases
AST	Asthma	Pulmonic diseases
BSR	Bipolar disorder	Psychiatric diseases
CAR	Cardiac disease	Cardiovascular diseases
COP	COPD / severe asthma	Pulmonic diseases
DEP	Depression	Psychiatric diseases
DM1	Type I Diabetes	Endocrine diseases
DM2	Type II Diabetes	Endocrine diseases
DMH	Diabetes with hypertension	Endocrine diseases
EPI	Epilepsy	Neurological diseases
GLA	Glaucoma	Other
HCH	High Cholesterol	Cardiovascular diseases
HIV	HIV / AIDS	Other
KHO	Hormone-sensitive Cancers	Cancer
KRE	Cancer	Cancer
KRK	Cancer complex	Cancer
MCR	Crohn's disease/ Ulcerative colitis	Autoimmune diseases
MSK	Multiple Sclerosis	Neurological diseases
NIE	Kidney diseases	Other
PAH	Hypertension	Cardiovascular diseases
PAR	Parkinson's disease	Neurological diseases
PSO	Psoriasis	Autoimmune diseases
PSY	Psychosis	Psychiatric diseases
RHE	Rheumatism	Other
SMC	Chronic pain without opioid use	Pain syndromes
SMN	Neuropathic pain	Pain syndromes
THY	Thyroid diseases	Endocrine diseases
TRA	Transplantation	Other
WAS	Growth disturbance	Other
ZFP	Cystic fibrosis / Pancreatic enzymes	Pulmonic diseases
ZNS	Central nerve system diseases excluding Multiple Sclerosis	Neurological diseases

Consumer Price Index (CPI) and Inflation rates (2015-2019)

Year	CPI[a]	Inflation rate[b]	Adjusting factor
2015	100		0.986186194
2016	99.56538	-0.43%	0.981900032
2017	100.09685	0.53%	0.987141315
2018	101.03409	0.94%	0.996384247
2019	101.40073	0.36%	1

Source: [a] Organization for Economic Co-operation and Development; Federal Statistical Office (FSO): Swiss Consumer Price Index (CPI) https://www.bfs.admin.ch/bfs/en/home/statistics/prices/consumer-price-index.html.

Exclusion codes:

Combination of Tarmed codes suggesting pathology not located in the lumbar spine but rather in the cervical spine [and adjacent skull base])

39.4100	CT Scan. Spine.	+ 39.4020 (CT neurocranium)<br>+ 39.4030 (CT skull)<br>+ 39.4050 (CT neck)<br>+ 39.4040 (CT dental area)<br>+ 39.4110 (CT shoulder)
39.5060	MRI Spine	+ 39.5050 (MRI neurocranium)<br>+ 39.5070 (MRI skull)<br>+ 39.5080 (neck)<br>+ 39.5140 (shoulder)

Tarmed codes for different operative procedures at the spine:

- 06.01 to 06.11

DRG-codes for an inpatient operative procedure at the spine:

B02B, B02C, B03B, B03C, B18Z, B20A, B20C, B20E, B61A, B61B, B61C, I06A, I06C, I09A, I09C, I09D, I09E, I10A, I10C, I45A, I45B, I68A, I68B, I68C, 68E, I68f

ATC Codes for i) pain medication and ii) other medications

1) Pain medications		
**NSAIDS (M01A)**		
	Celecoxib	M01AH01
	Diclofenac	M01AB05
	Diclofenac topical	M02AA15
	Diclofenac combinations	M01AB55
	Etodolac	M01AB08
	Ibuprofen	M01AE01
	Ibuprofen topical	M02AA13
	Ibuprofen combinations	M01AE51
	Ibuprofen + Oxicodone	N02AJ19
	Indomethacin	M01AB01
	Mefenamic Acid	M01AG01
	Naproxen	M01AE02
	Naproxen topical	M02AA12
	Naproxen + Esomeprazole	M01AE52
	Naproxen + Misoprostol	M01AE56
	Ketorolac	M01AB15
**Paracetamol**		
	Paracetamol	N02BE01
	Paracetamol	N02BE05
	Paracetamol combinations	N02BE71
**Opioids (N02A)**		
Weak Opioids	Dihydrocodeine	N02AA08
	Codeine (combinations)	N02AA59
	Tilidine	N02AX01
	Tramadol	N02AX02
	Tapentadol	N02AX06
	Tramadol combinations	N02AJ15
	Tramadol paracetamol	N02AJ13
Strong Opioids	Morphine	N02AA01
	Morphine combinations	N02AA51
	Hydromorphone	N02AA03
	Hydromorphone combinations	N02AA53
	Nicomorphine	N02AA04
	Oxycodone	N02AA05
	Oxycodone + ASS	N02AJ18
	Oxycodone + Ibuprofen	N02AJ19
	Oxycodone +Paracetamol	N02AJ17
	Oxycodone + Naloxon	N02AA55
	Pethidine	N02AB02
	Pethidine combinations	N02AG03
	Fentanyl systemic	N01AH01
	Fentanyl topic	N02AB03
	Fentanyl combinations	N01AH51
	Buprenorphine	N02AE01
	Nalbuphine	N02AF02
	Methadone combinations	N02AC52
	Piritramid	N02AC03

2) Co - medications		
**Muscle relaxants (M03)**		
Antispasticity medications	Baclofen	M03BX01
	Dantrolene	M03CA01
Antispasmodic medications	Metaxalone	
	Carisoprodol	M03BA02<br>M03BA52 (combinations)<br>M03BA72 (combinations)
	Chlorzoxazone	M03BB03 (single)<br>M03BB53 (combinations)<br>M03BB73 (combinations)
	Cyclobenzaprine	M03BX08
	Methocarbamol	M03BA03<br>M03BA53 (combinations)<br>M03BA73 (combinations)
	Orphenadrine	N04AB02 (chloride)<br>M03BC01 (citrate)<br>M03BC51 (combinations)
Antispasticity and Antispasmodic Agents	Tizanidine	M03BX02
	Diazepam	N05BA01
**Proton pump inhibitors (A02BC)**		
	Omeprazole	A02BC01
	Pantoprazole	A02BC02
	Lansoprazole	A02BC03
	Rabeprazole	A02BC04
	Esomeprazole	A02BC05
	Vonoprazan	A02BC08
**Laxatives**		
Drugs for constipation		A06A
Propulsives		A03F
**Sleeping pills**		
Sedating antihistamines	Diphenhydramine	R06AA02
	Doxylamine	R06AA09
	Cyclizine	R06AE03
Hypnotics	Zolpidem	N05CF02
	Zaleplon	N05CF03
	Eszopiclone	N05CF04
	Ramelteon	N05CH02
Benzodiazepines	Alprazolam	N05BA12
	Diazepam	N05BA01
	Lorazepam	N05BA06
	Triazolam	N05CD05
	Estazolam	N05CD04
	Temazepam	N05CD07
Tricyclic antidepressiva	Doxepin	N06AA12
other	Suvorexant	N05CM19
**Antidepressants (N06A)**		
	Amitriptyline	N06AA09
	Clomipramine	N06AA04
	Doxepin	N06AA12
	Imipramine	N06AA02
	Trimipramine	N06AA06
	Amoxapine	N06AA17
	Desipramine	N06AA01
	Nortriptyline	N06AA10
	Protriptyline	N06AA11
	Maprotiline	N06AA21

	Mirtazapine	N06AX11
	Trazodone	N06AX05
	Bupropion	N06AX12
	Venlafaxine	N06AX16
	Nefazodone	N06AX06
	Fluoxetine	N06AB03
	Paroxetine	N06AB05
	Sertraline	N06AB06
	Citalopram	N06AB04
	Fluvoxamine	N06AB08
	Isocarboxazid	N06AF01
	Phenelzine	N06AF03
	Tranylcypromine	N06AF04

Statistical models description

Details of regression models used

A multivariable mixed model with patients as random effects and correction for autocorrelation, ARMA(1,1) was implemented to study the association of patient variables with the overall costs.

Mixed model are required in our case because, for the same patient/insured, we have repeated measurements (from index year to two years after and one before). That means that our model should be corrected for correlated observations – within predictor variables (of course, observations from the same patient violated the hypothesis of independent observations in a simple regression model).

Therefore, we introduced random effects at patient levels. In particular, random effects were introduced as intercept: that is to say that each patient has its own intercept (in Figure 2 the fitted lines are reported as average between these random effects. Technically, we say we showed only the fixed effect part).

Moreover, we also considered an autocorrelation of type ARMA(1,1). This is a different type of correlation. In fact this is a correlation supposed for outcome and not for predictors. In other words, we supposed that the cost in the following year depended on the cost in the previous years. ARMA means autoregressive–moving-average. The value between parenthesis (1,1) means that we chose a model which regresses the variable on its own lagged, past values, of order 1 – that means outcome (costs) at a particular year is correlated with the outcome at the year before. The other value 1 means the order of the moving average: the error term of the model is as a linear combination of error terms occurring contemporaneously and at the year before. (In general, finding appropriate values of p and q in the ARMA(p,q) model can be facilitated by plotting the partial autocorrelation functions for an estimate of p, and likewise using the autocorrelation functions for an estimate of q).

A quadratic growth over time ($Time^2$+Time) was detected. In fact we verified that costs don't vary linearly over time, time being defined from index imaging year.

In details, the model was specified as follows:

Y ~ intercept + Fixed effects + [Random effect = patient]

Fixed effects = Age (continuous variable) + Gender (F/M)+ Medical services (yes/no) + Comorbidity (yes/no) + Area of residence (Abroad/German/Italian speaking) + Type of insurance (Network model/Family Physician model/Telemedicine model) + Deductible (≤500, 500-1500, ≤ 2500) + ($Time^2$+Time) + Index imaging year (2016/2017) + Type of insurance: Deductible.

The term *Type of insurance: Deductible* means the interaction effect between Type of insurance and deductible. The interaction terms Type of insurance: Deductible represented the type of insurance differences for each deductible.
For instance, the term Telemedicine model: deductible ≤ 500 estimated how much lower, in terms of costs, is the effect of telemedicine model for a deductible ≤ 500, compared with a free choice model. Interaction terms which are not significant means, graphically, that lines for different groups of type of insurance could look as "parallel" (or not interacted) on varying of deductible.
